# Chaiyin Granules Improve Acute Lung Injury by Regulating Gut Microbiota and Unsaturated Fatty Acid Mediated Wnt/β‐Catenin Signaling Pathway

**DOI:** 10.1155/jimr/7225946

**Published:** 2026-06-03

**Authors:** Wenting Ni, He Xiao, Qun Feng, Shirong Li, Jingchun Yao, Guimin Zhang

**Affiliations:** ^1^ College of Pharmacy, Shandong University of Traditional Chinese Medicine, Jinan, 250355, China, sdutcm.edu.cn; ^2^ State Key Laboratory of Integration and Innovation of Classic Formula and Modern Chinese Medicine, Lunan Pharmaceutical Group Co., Ltd., Linyi, 273400, China, lunan.com.cn; ^3^ Experimental Research Center, China Academy of Chinese Medical Sciences, Beijing, 100700, China, cacms.ac.cn

**Keywords:** acute lung injury, Chaiyin granules, gut microbiota, proteomics, unsaturated fatty acids, Wnt/β-catenin

## Abstract

**Aim of Study:**

This study aimed to investigate the efficacy and mechanism of Chaiyin granules (CY) in improving acute lung injury (ALI) based on the gut–lung axis.

**Materials and Methods:**

UHPLC‐ESI‐Q‐Exactive Orbitrap MS was used to analyze the active ingredients of CY. Establishment of lipopolysaccharide (LPS)–induced ALI mouse model and grouping: control, model, CYL (1.56 g/kg), CYM (3.12 g/kg), CYH (6.24 g/kg), and Dex (2 mg/kg) groups. The lung index and wet/dry (W/D) weight ratio of the lungs were calculated. The pathological damage of lung tissue was detected and evaluated using hematoxylin‐eosin (H&E) staining. The expression of various inflammatory factors and oxidative stress‐related factors was detected using cytometric bead array (CBA) and enzyme‐linked immunosorbent assay (ELISA), respectively. The indicators characterizing intestinal barrier damage were detected through the application of Alcian blue periodic acid Schiff (AB‐PAS) staining, ELISA, and western blot. Furthermore, gut microbiota were analyzed by 16S rRNA, and serum and lung metabolites were analyzed by metabolomics. Finally, proteomics was applied to enrich key signaling pathways, and western blot was used to validate the relevant proteins.

**Results:**

Fifty‐five active components in CY were identified. The pharmacological results showed that CY significantly alleviated lung pathological injury and inflammatory response of lung tissue in ALI mice while improving the integrity of intestinal structure and the permeability of the mucosal barrier in ALI mice. The evaluation was based on the improvement of abnormal expression of inflammatory factors, oxidative stress factors, tight junction proteins, goblet cells, LPS, secretory immunoglobulin A (SIgA), diamine oxidase (DAO), and D‐lactic acid (D‐LA), to a certain extent. Furthermore, 16S rRNA and metabolomics results showed that multiple core bacterial genera and unsaturated fatty acids were involved during CY treatment. Finally, proteomic results indicated that the Wnt/β‐catenin pathway was the mechanism of action for CY treatment of ALI, and CY effectively regulated the protein levels of Wnt3a, β‐catenin, and phosphorylated glycogen synthase kinase‐3β (p‐GSK3β).

**Conclusion:**

This study confirmed that CY could exert pharmacological effects on improving ALI mice through the gut–lung axis and also found that core bacterial genera and unsaturated fatty acids were involved in this process. More importantly, the Wnt/β‐catenin signaling pathway had been identified as a key signaling pathway for CY to alleviate ALI.

## 1. Introduction

Acute lung injury (ALI) is a sudden and severe inflammatory disease of the lungs, characterized by damage to alveolar epithelial cells, increased permeability of alveolar capillary membranes, and excessive inflammatory responses [1]. If left untreated, ALI may progress rapidly to respiratory failure and multiple organ dysfunction syndromes [2]. At present, glucocorticoids, neuromuscular blockers, and respiratory support are used, but the results are not ideal [3]. Therefore, there is an urgent need to find new drugs or treatment strategies for the improvement of ALI.

Nevertheless, the proposal of the gut–lung axis theory highlights the close crosstalk between the intestine and lungs [4]. As is well known, the gut and lungs are the first important immune barriers for the body to fight against foreign pathogens. They share a mucosal immune system and play an important role in regulating the body’s internal environment homeostasis and immune response through the interaction between the gut and lungs [5]. For example, researchers found that the level of gut microbiota in pneumonia after lung cancer surgery generally decreased, leading to an abnormal increase in the level of pathogenic microbiota [6]. In addition, a preclinical study found that secondary ALI was accompanied by the development of colitis, which explained the interconnectivity and true existence of the gut–lung axis [7]. More importantly, clinical studies had shown that abnormal increases in inflammatory factors in patients with ALI could damage the intestinal barrier function, leading to disturbances in the gut microbiota and metabolites that migrate into the bloodstream, ultimately exacerbating lung damage and inflammatory response [8]. Based on this, the gut–lung axis may be a novel strategy for treating lung injury by improving gut microbiota in the future.

Chaiyin granules (CY) are derived from the traditional Chinese medicine (TCM) formulas Chaige Jieji decoction and Yinqiao powder. Ingredients includes *Bupleurum chinense* DC. (Chaihu), *Lonicera japonica* Thunb. (Jinyinhua), *Scutellaria baicalensis* Georgi (Huangqin), *Pueraria lobata* (Willd.) Ohwi (Gegen), *Schizonepeta tenuifolia* (Benth.) Briq. (Jingjie), *Artemisia annua* L. (Qinghao), *Forsythia suspensa* (Thunb.) Vahl (Lianqiao), *Platycodon grandiflorus* (Jacq.) A. DC (Jiegeng), *Prunus armeniaca* L. var. *ansu* Maxim. (Kuxingren), *Mentha haplocalyx* Briq. (Bohe), and *Houttuynia cordata* Thunb. (Yuxingcao). In clinical practice, it was used to treat various lung diseases and inflammatory disorders [9, 10]. Chaihu can alleviate ALI by inhibiting the overactivation of complement and the production of proinflammatory mediators [11]; Jingyinhua can inhibit ferroptosis to alleviate ALI by activating the Nrf2/GPX4 axis [1]; Lianqiao could alleviate ALI by acting on PPAR‐γ/RXR‐α in the lung–gut axis [12]. Besides, Huangqin, Jingjie, Jiegeng, and Yuxingcao have all shown good inhibitory effects during the progression of lung injury [13–16]. Although CY has been proved to be effective in the treatment of ALI, the mediating role of the gut–lung axis has not been fully elucidated. Therefore, this study adopted a multiomics strategy for in‐depth mining and elucidation.

This study constructed an ALI mouse model by intratracheal injection of LPS and for the first time explored the improvement effect of CY on lung injury and intestinal function in ALI mice based on the gut–lung axis. Notably, 16S rRNA, metabolomics, and proteomics techniques were combined to elucidate the core bacterial genera and metabolites involved in CY’s improvement process of ALI, as well as key molecular mechanisms. These findings not only provide a new strategy for the treatment of ALI in clinical practice but also provide a solid scientific basis for CY treatment of ALI.

## 2. Materials and Methods

### 2.1. Drugs and Reagents

CY were supplied by Lunan Hope Pharmaceutical Co., Ltd. (National medicine approval Z20050338) (Linyi, China). Lipopolysaccharides (LPSs) were supplied from Sigma (United States). BD Pharmingen (CA, United States): mouse TNF‐α (Item Number 558299), IL‐1β (Item Number 560232), and IL‐6 (Item Number 558301) kits. D‐lactate kit (Item Number ml974725 L) was provided by Shanghai Enzyme Linked Biotechnology Co., Ltd. (Shanghai, China). The Alcian blue periodic acid Schiff (AB‐PAS) kit was provided by Solarbio (Item Number G1285) (Beijing, China). Cloud‐Clone Co., Ltd. (Wuhan, China): superoxide dismutase (SOD) (Item Number SES134Mu), diamine oxidase (DAO) (Item Number SEA656Mu), myeloperoxidase (MPO) (Item Number SEA601Mu) kits, LPS (Item Number SEB526Ge), and secretory Immunoglobulin A (SIgA) (Item Number SEA641Mu). Antibodies against β‐catenin (Item Number M24002) was sourced from Abmart Co., Ltd. (Shanghai, China). Antibodies against ZO‐1 (Item Number ab276131) and Occludin (Item Number ab216327) were obtained from Abcam (Cambs, UK). Nanjing Jiancheng Bioengineering Institute (Nanjing, China): malondialdehyde (MDA) (Item Number A003‐1‐2) kits, glutathione (GSH) (Item Number A006‐2‐1), catalase (CAT) (Item Number A007‐1‐1). Anti‐Wnt3a (Item Number 2721), GSK‐3β (Item Number 12456), and phospho‐GSK‐3β (Item Number 5558) antibody were purchased from CST (BSN, MA, USA). Anti‐GAPDH (Item Number AF2819) antibody was obtained from Beyotime Technology (Shanghai, China). Dexamethasone (Dex) sodium phosphate injection was supplied from Chenxin Pharmaceutical Co. Ltd. (National medicine approval H53020638) (Jining, China).

### 2.2. Animals

Forty‐eight male C57BL/6J mice (7–8 weeks old, 18–20 g body weight) were randomly divided into six groups according to body weight (*n* = 8 per group): control, model, CYL‐1.56 g/kg, CYM‐3.12 g/kg, CYH 6.24 g/kg group, and Dex‐2 mg/kg group, and the animals were provided by Jinan Pengyue Co., Ltd. (Quality Certificate Number: SCXK Lu 2022 0006). All animal breeding and experimental procedures were reviewed and approved by the Experimental Animal Ethics Committee of the State Key Laboratory of Integration and Innovation of Classic Formula and Modern Chinese Medicine (Ethics Approval Number HA‐IACUC‐2024‐127).

### 2.3. UHPLC‐ESI‐Q‐Exactive Orbitrap MS Analysis

The components of CY were detected by UHPLC‐ESI‐QE Orbitrap MS. The detailed procedures and experimental conditions were performed as previously described [17].

### 2.4. Induction and Intervention in ALI Mice

ALI mouse models were constructed according to reported studies [18]. In this study, the ALI model was established by intratracheal injection of LPS (5 mg/kg), while the control group was injected with normal saline. According to the procedure of a previously published study [17], before intratracheal infusion of LPS, the CY group was given oral CY for 7 days, and the Dex group was given an intraperitoneal injection of Dex for 3 days. All treatments were administered once daily. The control group and the model group were given the same amount of pure water orally. At 24 h after LPS injection (only once intratracheally), mice were anesthetized using a mixture of ketamine hydrochloride (7.5 mg/kg) and xylazine hydrochloride (7.5 mg/kg), while serum, lung tissue, colon, and feces were collected for subsequent studies.

### 2.5. Lung Index and Wet/Dry (W/D) Lung Weight Ratio

Lung wet (fresh tissue)/dry (oven‐dried tissue) weight (W/D) ratio was assessed. The lung index was evaluated before drying, according to the following formula:

Lung index=lung weight gbody weight g×100%.

### 2.6. Hematoxylin‐Eosin (H&E) Staining

Gut and lung tissues from each group of mice were stored in a tissue fixative solution. Then, the fixed samples were placed sequentially in different concentrations of ethanol solutions for dehydration. Paraffin sections were subsequently made. The prepared sections were subjected to H&E operations. The changes of the tissues were scored by pathologists using light microscopy.

### 2.7. Cytometric Bead Array (CBA)

Supernatants of serum and lung tissues were prepared by centrifugation. According to the instructions provided by the reagent manufacturer, all inflammatory factors were detected by the CBA technology.

### 2.8. Oxidative Stress Factor Assay

The supernatant of lung tissues was obtained. DAO (SOD), CAT, reduced glutathione, MPO, SIgA, and MDA were detected according to the instructions of the kit.

### 2.9. Analysis of LPSs, D‐Lactate, and DAO

The contents of LPS, D‐LA, and DAO in serum were detected according to the instructions provided by the reagent manufacturer.

### 2.10. AB‐PAS Staining

The changes in intestinal goblet cells were evaluated using AB‐PAS staining. Neutral mucins are colored magnetic, whereas acidic mucins are colored blue. In brief, prepared colon sections were infiltrated with Alcian blue for 15 min. Then, the samples were stained with Schiff’s reagent for an additional 15 min. Finally, ImageJ was applied to calculate the number of goblet cells and to observe the morphology.

### 2.11. 16S rRNA

Combined with the results of animal experiments, the high‐dose group of CY had the best protective effect in pharmacodynamic indicators, so the high‐dose group was used for follow‐up mechanism research. Genomic DNA was extracted from feces samples and tested simultaneously for purity and concentration. The PCR products obtained were examined. The library quality and quantification were performed by NanoDrop and Qubit, respectively. The qualified libraries were sequenced using the Illumina HiSeq 2500 high‐throughput sequencing platform. OTU analysis and species annotation were performed based on a 97% sequence identity level. Alpha and beta diversity were calculated based on OTU. The Wekemo Bioincloud scientific research platform was used to visualize the stacked bar chart of species percentage. Linear discriminant analysis effect size (LEfSe) analysis was used to find significant biomarkers with the criteria of *p* < 0.05 and LDA > 2.

### 2.12. Metabolomics

The metabolic profile of serum and lung tissues in each group was analyzed by nontargeted metabolomics. The detailed procedure was referred to the published study [17]. Simca‐p 14.0 software was used for principal component analysis (PCA) and orthogonal partial least‐squares discriminant analysis (OPLS‐DA) analysis. The MetaboAnalyst 6.0 online platform was used to analyze the metabolic pathways of differential metabolites in lung tissue and serum to find the key metabolic markers. Spearman correlation was used to analyze the correlation between metabolic markers and core bacterial genera.

### 2.13. Proteomics

The proteins from the intestinal and lung tissues were extracted, and their concentrations were determined. Subsequently, all the samples were subjected to acetone precipitation, protein resolubilization, reduction, alkylation, proteolysis, removal of SDC, and peptide desalting. Finally, all the samples were analyzed using nanoliquid chromatography combined with tandem mass spectrometry (LC‐MS/MS), and the data were analyzed.

### 2.14. Western Blotting

Tissue protein supernatants were obtained from each group of mice. The OD value at 562 nm wavelength was detected by a microplate reader to draw the standard curve, and the protein concentration of each group was calculated. Subsequently, electrophoresis, membrane transfer, and blocking of the proteins were performed. Further, primary and secondary antibody solutions were incubated. Finally, the ChemiScope 6200 chemiluminescence imager was used for image processing. ChemiScope Analysis software was detected to analyze the gray value of proteins.

### 2.15. Statistical Analysis

Data were expressed as mean ± standard deviation (SD). All statistical analyses were performed using SPSS 19.0. Comparisons between two groups were conducted using Student’s *t*‐test. For multiple group comparisons, one‐way analysis of variance (ANOVA) was employed, followed by Tukey’s honest significant difference post hoc test. A *p* < 0.05 was considered statistically significant.

## 3. Results

### 3.1. Chemical Composition Analysis of CY

**Figure 1:**
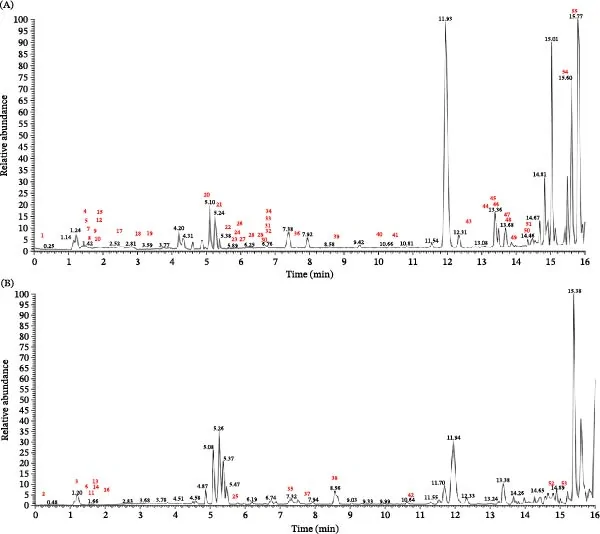
A total of 55 components of CY were identified by the UHPLC‐ESI‐QE Orbitrap MS. (A) Positive and (B) negative ion mode.

Fifty‐five active ingredients of CY were detected in both positive (Figure 1A) and negative (Figure 1B) ion modes. The information is shown in Table 1.

**Table 1:** The components in CY.

Number	Name	Formula	m/z	*R*_*T*_ (min)	Reference ion
1	Eucalyptol	C_10_H_18_O	137.133	0.132	[M + H] + 1
2	Malonic acid	C_3_H_4_O_4_	103.0036	0.293	[M−H] − 1
3	β‐N‐oxalyl‐α, β‐diaminopropionic acid	C_5_H_8_N_2_O_5_	174.9559	1.28	[M−H] − 1
4	(−)‐Camphor	C_10_H_16_O	153.1262	1.404	[M + H] + 1
5	Acetophenone	C_8_H_8_O	121.0643	1.431	[M + H] + 1
6	Oxalic acid	C_2_H_2_O_4_	88.98801	1.457	[M−H] − 1
7	L‐(−)‐serine	C_3_H_7_NO_3_	147.0765	1.462	[M + ACN + H] + 1
8	L‐(−)‐threonine	C_4_H_9_NO_3_	120.0658	1.498	[M + H] + 1
9	L‐pyroglutamic acid	C_5_H_7_NO_3_	147.0765	1.515	[M + NH_4_] + 1
10	Betaine	C_5_H_11_NO_2_	118.0866	1.525	[M + H] + 1
11	Purine	C_5_H_4_N_4_	101.0252	1.58	[M–H–H_2_O] − 1
12	5‐Hydroxymethylfurfural	C_6_H_6_O_3_	127.0392	1.584	[M + H] + 1
13	α,α‐Trehalose	C_12_H_22_O_11_	377.0852	1.607	[M + Cl] − 1
14	Sucrose	C_12_H_22_O_11_	377.0908	1.629	[M + Cl] − 1
15	DL‐glutamine	C_5_H_10_N_2_O_3_	147.0765	1.643	[M + H] + 1
16	L‐(+)‐lactic acid	C_3_H_6_O_3_	89.02432	2.022	[M − H] − 1
17	L‐(+)‐leucine	C_6_H_13_NO_2_	132.1021	2.506	[M + H] + 1
18	Isothujone	C_10_H_16_O	153.1272	3.053	[M + H] + 1
19	3‐Phenylpropanoic acid	C_9_H_10_O_2_	301.1441	3.458	[2M + H] + 1
20	Indole	C_8_H_7_N	118.0655	5.086	[M + H] + 1
21	n‐Hexanamide	C_6_H_13_NO	116.1076	5.268	[M + H] + 1
22	Tryptamine	C_10_H_12_N_2_	161.1075	5.549	[M + H] + 1
23	Benzoxazole	C_7_H_5_NO	120.0447	5.75	[M + H] + 1
24	Allyl propyl disulfide	C_6_H_12_S_2_	190.0714	5.822	[M + ACN + H] + 1
25	Azelaic acid	C_9_H_16_O_4_	187.0978	5.829	[M − H] − 1
26	Benzaldehyde	C_7_H_6_O	124.0757	5.918	[M + NH_4_] + 1
27	Valerophenone	C_11_H_14_O	204.1385	5.998	[M + ACN + H] + 1
28	Benzothiazole	C_7_H_5_NS	136.0218	6.38	[M + H] + 1
29	Isophorone	C_9_H_14_O	139.112	6.593	[M + H] + 1
30	A‐terpineol	C_10_H_18_O	172.1697	6.635	[M + NH_4_] + 1
31	Cinnamaldehyde	C_9_H_8_O	133.0649	6.645	[M + H] + 1
32	Benzyl isothiocyanate	C_8_H_7_NS	150.0375	6.647	[M + H] + 1
33	Palmitic acid	C_16_H_32_O_2_	274.2745	6.698	[M + H] + 1
34	Ionene	C_13_H_18_	175.1482	6.738	[M + H] + 1
35	Recifeiolide	C_12_H_20_O_2_	195.1389	7.32	[M − H] − 1
36	Nootkatone	C_15_H_22_O	219.1744	7.585	[M + H] + 1
37	Deoxycholic acid	C_24_H_40_O_4_	391.2855	7.774	[M − H] − 1
38	L‐tyrosine methyl ester	C_10_H_13_NO_3_	194.0821	8.565	[M − H] − 1
39	D‐sphingosine	C_18_H_37_NO_2_	300.2898	8.775	[M + H] + 1
40	Myristamide	C_14_H_29_NO	228.2325	10.063	[M + H] + 1
41	Furfuranol	C_5_H_6_O_2_	137	10.549	[M + K] + 1
42	Fusaricate I	C_14_H_21_NO_3_	250.1448	10.649	[M − H] − 1
43	Phenethylamine	C_8_H_11_N	122.0969	12.782	[M + H] + 1
44	Pentyl isothiocyanate	C_6_H_11_NS	130.0684	13.19	[M + H] + 1
45	γ‐Linolenic acid ethyl ester	C_20_H_34_O_2_	313.274	13.378	[M + H] + 1
46	Ethyl myristate	C_16_H_32_O_2_	239.2378	13.415	[M +H–H_2_O] + 1
47	DL‐α‐tocopherol	C_29_H_50_O_2_	431.3846	13.777	[M + H] + 1
48	Cytosine	C_4_H_5_N_3_O	134.0322	13.832	[M + Na] + 1
49	Ligustrazine	C_8_H_12_N_2_	137.1083	14.08	[M + NH_4_] + 1
50	Stearamide	C_18_H_37_NO	284.2951	14.348	[M + H] + 1
51	Erucamide	C_22_H_43_NO	338.3434	14.358	[M + H] + 1
52	2‐Ethyl‐4,5,6,7‐tetraiodo‐1H‐benzimidazole	C_9_H_6_I_4_N_2_	648.6614	14.784	[M − H] − 1
53	Cyathisterone	C_28_H_42_O_2_	409.3105	15.218	[M − H] − 1
54	5‐Methyl‐3‐isoxazolamine	C_4_H_6_N_2_O	99.05569	15.562	[M + H] + 1
55	Hexadecanamide	C_16_H_33_NO	256.2634	15.781	[M + H] + 1

### 3.2. Pharmacodynamic Effects of CY on ALI Mice

**Figure 2:**
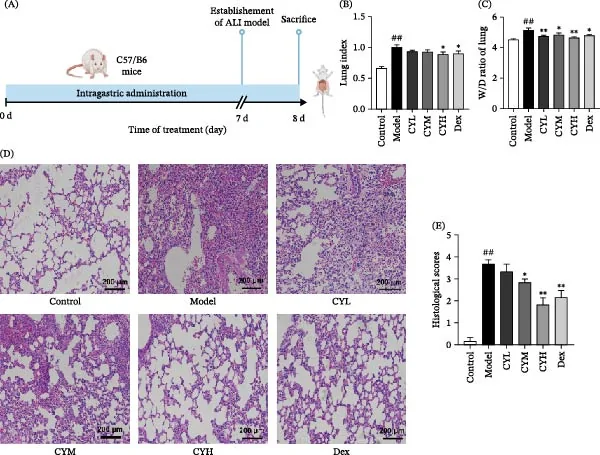
The protective effect of CY in ALI mice. (A) Experimental process diagram. (B) Lung index (*n* = 8 per group). (C) W/D ratio (*N* = 8 per group). (D) Pathological visualization of the lungs. (E) Histological scores of lung injury (*n* = 3 per group). ^##^ *p* < 0.01 vs. control group;  ^∗^ *p* < 0.05,  ^∗∗^ *p* < 0.01 vs. model group.

The entire process of experimental operation is shown in Figure 2A. The pharmacodynamic results showed that LPS could significantly increase the lung index of ALI mice (Figure 2B) and significantly increase the W/D ratio of lung tissues (Figure 2C), and these abnormalities were significantly reversed by CY treatment. Pathological damage: The lung lobule structure of the control group mice was relatively intact, without any infiltration of inflammatory cells, tissue edema, or bleeding; however, the model group mice showed significant infiltration of inflammatory cells, damage to lung lobular structures, and significant pulmonary hemorrhage and edema. Notably, lung pathological injury was markedly alleviated in the CYM, CYH, and Dex groups. The CYH group exhibited the most significant improvement, with histological scores even lower than those in the Dex‐positive control group (Figure 2D,E). Based on this, it indicated that CY could effectively improve LPS‐induced ALI.

### 3.3. Ameliorating Effects of CY on Inflammatory Response and Oxidative Stress in ALI Mice

**Figure 3:**
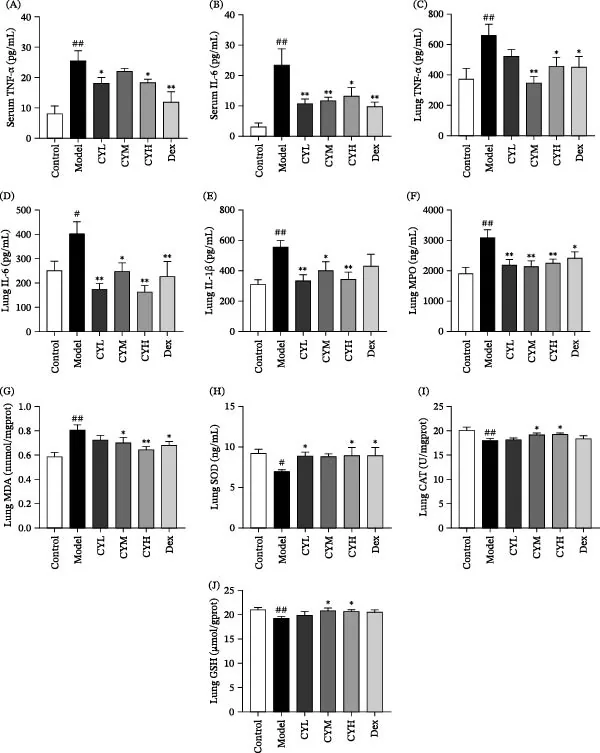
Effects of CY on inflammation and oxidative stress (*n* = 6 per group). (A) TNF‐α and (B) IL‐6 in serum. (C) TNF‐α, (D) IL‐6, and (E) IL‐1β in lung tissue. (F) MPO, (G) MDA, (H) SOD, (I) CAT, and (J) GSH in lung tissue. ^#^ *p* < 0.05, ^##^ *p* < 0.01 vs. control group;  ^∗^ *p* < 0.05,  ^∗∗^ *p* < 0.01 vs. model group.

Under oxidative stress, excessive free radicals produced could attack unsaturated fatty acids on the cell membrane, affecting the function of alveolar epithelial cells, leading to lung injury. In addition, oxidative stress could exacerbate lung injury by promoting the expression of various inflammatory factors [19]. Firstly, CBA technology was used to detect the expression of inflammatory cytokines in the serum and lung tissues of mice in each group. The results showed that the levels of TNF‐α (Figure 3A) and IL‐6 (Figure 3B) in the serum of ALI mice were significantly increased, while the levels of TNF‐α (Figure 3C), IL‐6 (Figure 3D), and IL‐1β (Figure 3E) in the lung tissue were also significantly increased, indicating an excessive inflammatory response in ALI mice. However, after the intervention of CY and Dex, the expression of these factors in the serum and lung tissues of the treated mice was decreased to varying degrees, and the effect of CYH was comparable to that of the Dex group in lung tissues, indicating that CY reduced the inflammatory response in the mice with ALI.

Furthermore, factors related to oxidative stress were detected in lung tissue. The levels of MPO (Figure 3F) and MDA (Figure 3G) in the lung tissue of the model group mice increased abnormally, while the levels of SOD (Figure 3H), CAT (Figure 3I), and GSH (Figure 3J) decreased abnormally. However, CY could significantly reduce the expression levels of these factors, while there was no significant difference in the expression levels of CAT and GSH in the Dex group. These results indicated that CY had a certain improvement effect on oxidative stress in ALI mice.

### 3.4. The Improvement Effect of CY on Intestinal Barrier Injury in ALI Mice

**Figure 4:**
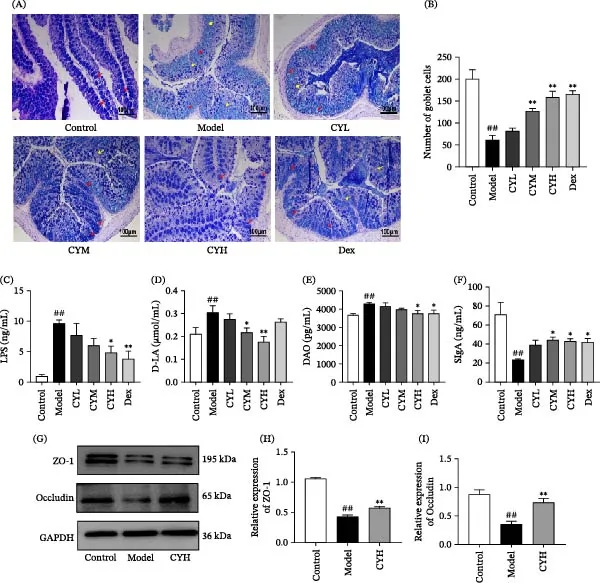
The protective effect of CY on the intestinal of ALI mice. (A) AB‐PAS. The red arrow indicated goblet cells; the yellow arrow indicated inflammatory cells. The shade of color (blue) in the particles within the cells indicated the degree of mucus secretion. (B) Number of goblet cells (*n* = 3 per group). (C) LPS, (D) D‐LA, and (E) DAO. (F) SIgA in colon (*n* = 6 per group). (G–I) ZO‐1 and Occludin proteins (*n* = 3 per group). ^#^ *p* < 0.05, ^##^ *p* < 0.01 vs. control group;  ^∗^ *p* < 0.05,  ^∗∗^ *p* < 0.01 vs. model group.

Studies have confirmed that ALI is often accompanied by the disruption of the intestinal barrier, which can lead to a significant increase in intestinal mucosal permeability and abnormal expression of tight junction proteins that maintain the structure of the mucosal barrier. Furthermore, harmful intestinal bacteria and their toxic metabolites and factors enter the bloodstream and exacerbate the original lung damage [20, 21]. Based on this, this study evaluated the improvement effect of CY on the intestinal barrier and the intestinal mucosa in ALI mice. The state of goblet cells is often used to assess the degree of intestinal mucosal damage by applying the AB‐PAS technique. The results showed that in the control group, the goblet cells in the mucus layer were plump and oval‐shaped, with no damage or injury. The goblet cells in the mice were reduced in the model group, accompanied by morphological deformation. Mucus secretion was significantly reduced, and inflammatory cell infiltration was visible (Figure 4A, B). However, in the CY group, the number of intestinal goblet cells was significantly increased in a dose‐dependent manner, and the effect of CYH was similar to that of the Dex group. Further, we detected the toxic metabolites (LPS, D‐LA, and DAO) derived from the microbiota. Generally, the levels of LPS (Figure 4C), D‐LA (Figure 4D), and DAO (Figure 4E) in the ALI mice were increased, and CY and Dex treatment significantly reduced the levels of these mediators. SIgA is an important substance for intestinal mucosal immunity that can resist pathogens [22]. The results in Figure 4F show that the SIgA level in ALI mice was reduced, while these abnormalities were significantly restored by CY and Dex, and CY showed comparable efficacy to Dex. Finally, western blotting was used to detect the expression of intestinal ZO‐1 and Occludin. The results showed that CY could upregulate the expression levels of ZO‐1 and Occludin in the intestinal mucosa of ALI mice (Figure 4G–I). In conclusion, CY had a significant protective effect on the damage of the intestinal barrier in ALI mice and provided a basis for the cross‐talk between the intestine and the lungs.

### 3.5. The Regulatory Effect of CY on the Gut Microbiota Imbalance in ALI Mice

The diversity and distribution characteristics of the gut microbiota were detected through the α and β diversity indicators in the 16S rRNA technology. The α diversity indicators (Ace, Chao, Sobs, and Shannon) in the ALI mice were lower compared to the control group, and this trend could be reversed after CY intervention (Figure 5A–D). The β diversity indicators (PCA and PCoA) showed significant differences and separations among the three groups of mice (Figure 5E,F). Additionally, the composition of the gut microbiota in each group of mice at the phylum and genus levels was analyzed, revealing that the control group and the CY group had more similar microbiota structures (Figure 5G,H). Furthermore, at the genus level, significant differences and rebounds were observed in the levels of *Ligilactobacillus*, *Parabacteroides*, and *Lachnospiraceae* among the three groups. These genera were identified as the core genera. Compared with the control group, the abundances of *Ligilactobacillus* (Figure 5I) and *Parabacteroides* (Figure 5K) were significantly increased, while the abundance of *Lachnospiraceae* (Figure 5J) was decreased. However, after CY intervention, the abnormal levels of these genera were restored. These results indicate that CY could balance the gut microbiota disorder in ALI mice.

Figure 5The regulatory effect of CY on the gut microbiota of ALI mice (*n* = 6 per group). (A–D) The α diversity of Ace, Chao, Shannon, and Sobs. (E, F) The β diversity of PCA and PCoA. (G, H) Constituent ratios of gut microbiota at phylum and genus levels. (I–K) The abundance of *Ligilactobacillus*, *Lachnospiraceae*, and *Parabacteroides*. (L) LEfSe. ^#^ *p* < 0.05, ^##^ *p* < 0.01 vs. control group;  ^∗^ *p* < 0.05,  ^∗∗^ *p* < 0.01 vs. model group.

**Figure:**
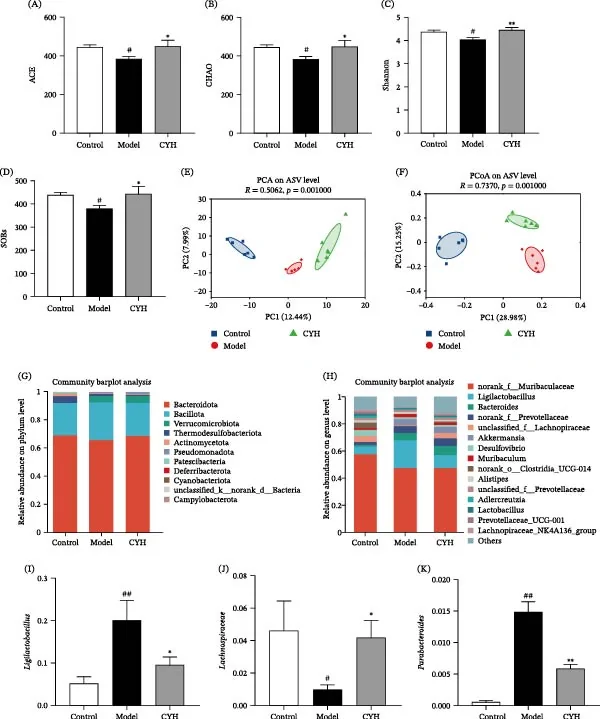

**Figure:**
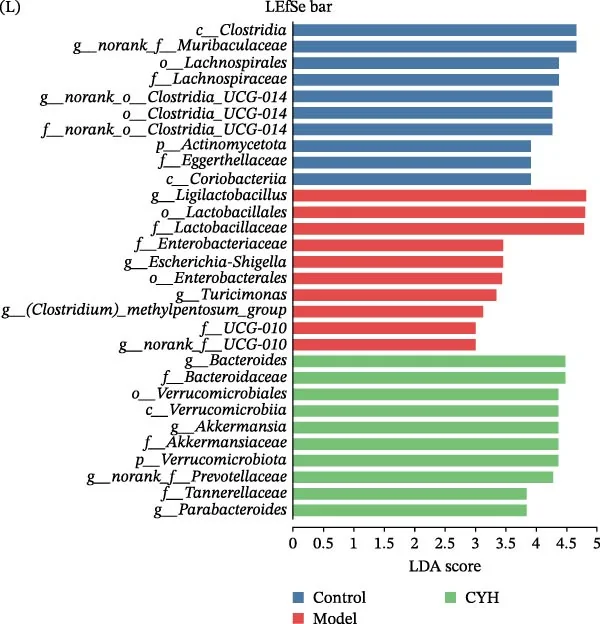

The LEfSe displayed that the abundance of *c_Clostridia*, *g_norank_f_Muribaculaceae*, *o_Lachnospirales*, and *f_Lachnospiraceae* was high in the control mice (Figure 5L). The abundance of *g_Ligilactobacillus*, *o_Lactobacillales*, and *f_Lactobacillaceae* exhibited high levels in the CYH group. In conclusion, CY could exert a protective effect on ALI mice by regulating the gut microbiota.

### 3.6. The Improvement Effect of CY on Metabolic Disorders in the Serum and Lung Tissue of ALI Mice

**Figure 6:**
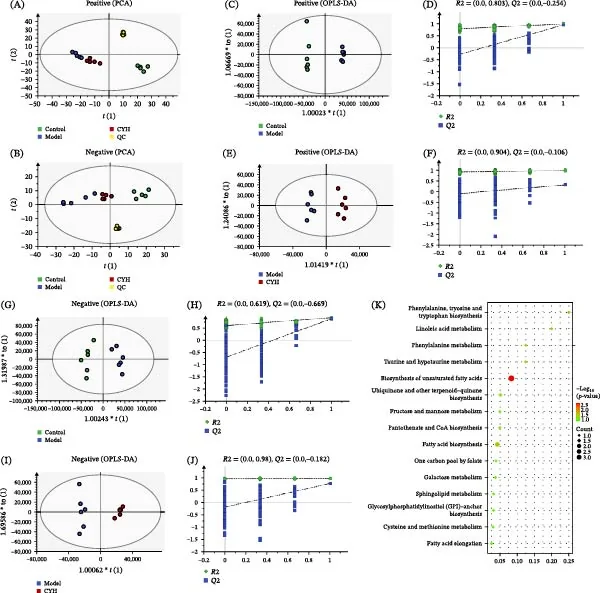
Analysis results of serum metabolomics of each group of mice (*n* = 6 per group). (A, B) PCA. (C–J) OPLS‐DA and permutation test. (K) KEGG pathway.

To validate the gut–lung axis, metabolic profiles of serum and lung tissues were examined. Firstly, serum metabolomics PCA and OPLS‐DA results showed a clear separation between the three groups. More importantly, the characteristics of the CYH group were more similar to those of the control group, indicating that CY could interfere with serum metabolic changes in ALI mice (Figure 6A–C,E,G,I). The reliability of OPLS‐DA results was further supported and verified by the permutation test (Figure 6D–F,H,J). *p* < 0.05 and VIP > 1.0 were used as screening conditions for differential metabolites in the three groups, and a total of 42 differential metabolites were screened (Supporting Information 1). Metabolic pathway enrichment mainly focused on phenylalanine, tyrosine, and tryptophan biosynthesis; linoleic acid metabolism; phenylalanine metabolism; and unsaturated fatty acid biosynthesis (Figure 6K and Table 2). These results suggested that multiple metabolic pathways mediated by a variety of serum metabolites were involved in the anti‐ALI process of CY.

**Table 2:** Metabolic pathway of serum.

Number	Metabolic pathway	Enrichment	Counts	Hits
1	Phenylalanine, tyrosine, and tryptophan biosynthesis	0.250	1	Tyrosine
2	Linoleic acid metabolism	0.200	1	Linoleate
3	Phenylalanine metabolism	0.125	1	L‐tyrosine
4	Taurine and hypotaurine metabolism	0.125	1	Taurocholate
5	Biosynthesis of unsaturated fatty acids	0.083	3	Hexadecanoic acid, linoleate, (6Z,9Z,12Z)‐octadecatrienoic acid
6	Ubiquinone and other terpenoid‐quinone biosynthesis	0.053	1	L‐tyrosine
7	Fructose and mannose metabolism	0.050	1	Pantothenate
8	Pantothenate and CoA biosynthesis	0.050	1	D‐mannose
9	Fatty acid biosynthesis	0.043	2	Hexadecanoic acid, dodecanoic acid
10	One carbon pool by folate	0.038	1	L‐methionine
11	Galactose metabolism	0.037	1	D‐mannose
12	Sphingolipid metabolism	0.031	1	Hexadecanoic acid
13	Glycosylphosphatidylinositol (GPI)‐anchor biosynthesis	0.031	1	Phytosphingosine
14	Cysteine and methionine metabolism	0.030	1	L‐methionine
15	Fatty acid elongation	0.026	1	Hexadecanoic acid

**Figure 7:**
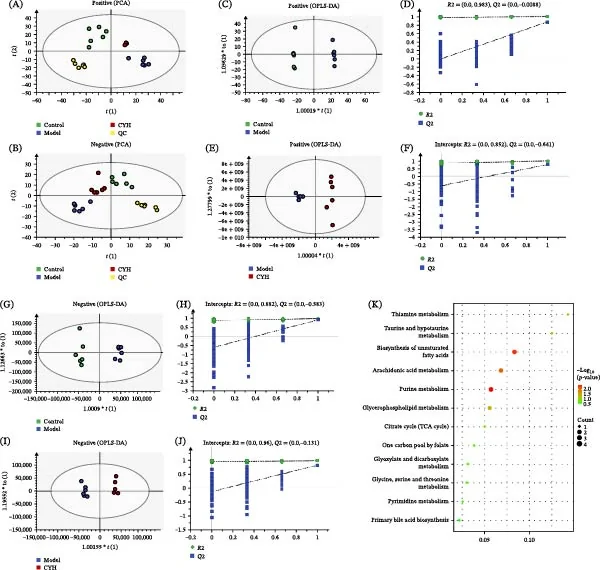
Metabolomics analysis results of lung tissues from each group of mice (*n* = 6 per group). (A, B) PCA. (C–J) OPLS‐DA and permutation test. (K) KEGG pathway.

Similar changes were observed in lung metabolomic results compared to serum metabolomic results (Figure 7A–J). A total of 58 differential lung tissue metabolites were screened (Supporting Information 2). The KEGG pathways included thiamine metabolism, biosynthesis of unsaturated fatty acids, taurine and hypotaurine metabolism, and arachidonic acid metabolism (Figure 7K). These results suggested that multiple metabolic pathways mediated by various lung tissue metabolites were involved in the anti‐ALI process of CY (Table 3).

**Table 3:** Metabolic pathway of lung tissues.

Number	Metabolic pathway	Enrichment	Counts	Hits
1	Thiamine metabolism	0.143	1	Thiamine
2	Taurine and hypotaurine metabolism	0.125	1	Taurine
3	Biosynthesis of unsaturated fatty acids	0.083	3	Octadecanoic acid, Arachidonate, Dihomo‐gamma‐linolenate
4	Arachidonic acid metabolism	0.068	3	Arachidonate, prostaglandin I2, 12(S)‐HETE
5	Purine metabolism	0.057	4	Xanthine, AMP, hypoxanthine, urate
6	Glycerophospholipid metabolism	0.056	2	1‐Acyl‐sn‐glycero‐3‐phosphocholine, choline
7	Citrate cycle (TCA cycle)	0.050	1	Isocitrate
8	One carbon pool by folate	0.038	1	Choline
9	Glyoxylate and dicarboxylate metabolism	0.031	1	Isocitrate
10	Glycine, serine, and threonine metabolism	0.030	1	Choline
11	Pyrimidine metabolism	0.026	1	CMP
12	Primary bile acid biosynthesis	0.022	1	Taurine

### 3.7. Correlation Analysis of Key Bacterial Genera and Core Metabolites

**Figure 8:**
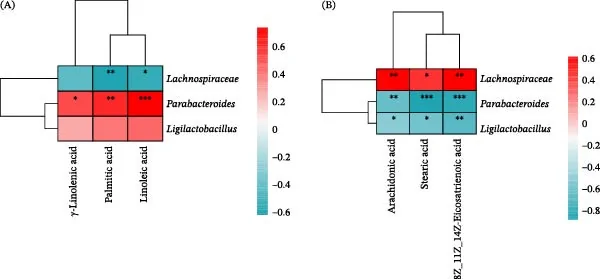
Correlation visual analysis. (A) Correlation visualization diagram (the genus of bacteria and serum metabolites). (B) Correlation visualization diagram (the genus of bacteria and lung metabolites).  ^∗^ *p* < 0.05,  ^∗∗^ *p* < 0.01, and  ^∗∗∗^ *p* < 0.001.

Based on the above metabolomics results, the biosynthesis of the unsaturated fatty acid metabolic pathway was considered to have played an important role in the CY anti‐ALI. Therefore, the key metabolites mapped to this pathway were subjected to correlation analysis with the aforementioned core bacterial genera. Specifically, palmitic acid, γ‐linolenic acid, and linoleic acid in serum were positively correlated with *Parabacteroides*, while palmitic acid and linoleic acid in serum were negatively correlated with *Ligilactobacillus* (Figure 8A). 8Z, 11Z, 14Z‐eicosatrienoic acid, stearic acid, and arachidonic acid in lung tissues were positively correlated with *Lachnospiraceae* and negatively correlated with *Parabacteroides* and *Ligilactobacillus* (Figure 8B). These results indicated a close relationship among the intestinal bacterial genera, serum metabolites, and lung metabolites.

### 3.8. Exploration of the Molecular Mechanism of CY in Improving ALI

**Figure 9:**
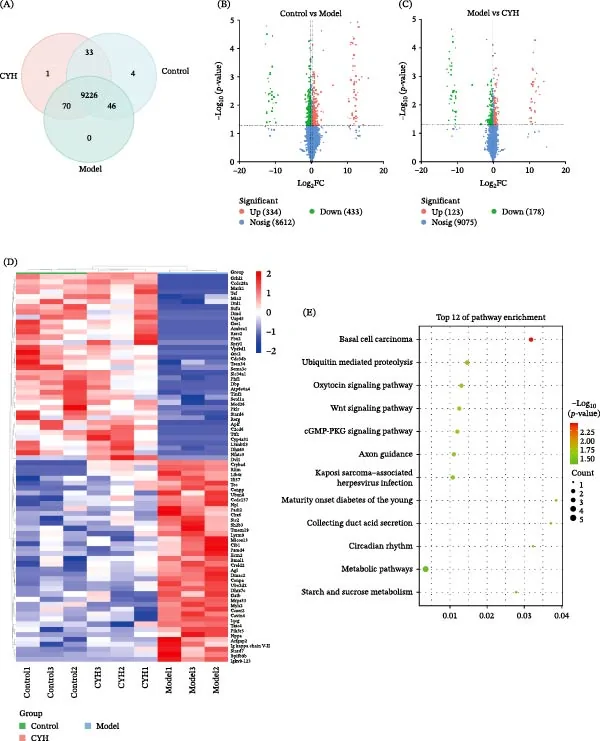
Proteomics results (*n* = 3 per group). (A) Venn diagram. (B, C) Volcano plot. (D) Heatmap. (E) KEGG analysis.

The proteomics technique was employed to explore the molecular mechanism by which CY improves ALI. The Venn diagram showed that there were a total of 9226 protein types among the three groups (Figure 9A). Additionally, the screening conditions for differentially expressed proteins were fold change > 1.2 or <0.83 and *p*  < 0.05. The volcano plot is shown in Figure 9B,C. Compared with the control group, a total of 334 proteins were upregulated, while 443 proteins were downregulated. However, compared with the model group, a total of 123 proteins were upregulated and 178 proteins were downregulated in the CYH group. Furthermore, 77 of the same proteins were significantly enriched among the three groups and were restored to normal levels by CY (Figure 9D). The KEGG results indicated that the signaling pathways mainly involved basal cell carcinoma, ubiquitin‐mediated protein degradation, Wnt signaling pathway, and GMP‐PKG signaling pathway (Figure 9E). More importantly, the Wnt signaling pathway was involved in ALI progression [23]. This suggested that the Wnt pathway was the key pathway of CY in anti‐ALI.

### 3.9. The Effect of CY on the Wnt/β‐Catenin Pathway in the Lung Tissue of ALI Mice

**Figure 10:**
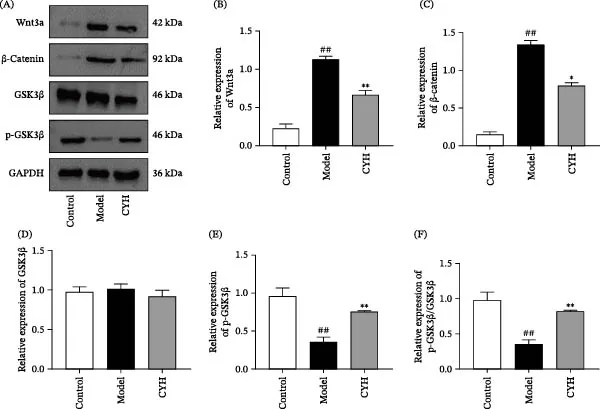
The effect of CY on the Wnt signaling pathway in ALI mice (*n* = 3 per group). (A–F) The levels and quantification of Wnt3a, β‐catenin, GSK3β, and p‐GSK3β. ^##^ *p* < 0.01 vs. control group;  ^∗^ *p* < 0.05,  ^∗∗^ *p* < 0.01 vs. model group.

Compared with the control group, the contents of Wnt3a and β‐catenin were increased in the model group, while the expression of the phosphorylated glycogen synthase kinase‐3β (p‐GSK3β) protein was decreased (Figure 10A–F). Surprisingly, these abnormal protein levels could be reversed to normal levels by CY, which proved that the Wnt signaling pathway played a crucial role in CY’s anti‐ALI effect.

## 4. Discussion

Numerous studies had demonstrated that the pathogenesis of ALI was closely related to inflammation, oxidative stress, intestinal flora imbalance, and metabolic disorders [21, 24, 25]. At present, effective and safe drugs still face significant challenges and shortages. TCM prescriptions, based on their own unique advantages and characteristics, had been widely recognized and applied by clinical experts. As CY, which has been widely applied in respiratory system diseases, its pharmacological mechanism against ALI has not been fully elucidated. In the previous study, we screened the doses of CY; after comprehensive inspection and evaluation of the experimental results, we found that the other doses were too not appropriate to achieve a good therapeutic effect. Finally, we selected 0.5, 1, and 2 times the clinical dose for research, namely, 1.56, 3.12, and 6.24 g/kg of CY groups. The preliminary pharmacodynamic results showed that CY could protect the lung injury in LPS‐induced ALI mice. Studies had confirmed that LPS could induce the body to trigger excessive inflammatory responses and oxidative stress [26–28]. Furthermore, the released inflammatory factors will act as initiating factors to exacerbate the original oxidative stress response, leading to further deterioration of lung damage. However, CY significantly suppressed the overproduction of these proinflammatory cytokines, which also indicates that CY has a certain anti‐inflammatory effect on ALI mice.

TNF‐α, IL‐1β, and IL‐6 have been proven to be the core driving factors of the inflammatory cascade in ALI [18, 29]. In the LPS‐induced ALI model, TNF‐α reached its peak at 30 min after injury, earlier than the peak of pathological damage, suggesting its value as an early warning indicator. Recent studies had found that TNF‐α could also reprogram the physiological autophagy of macrophages into caspase‐8–dependent pyroptosis through the TIM3 receptor, leading to the maturation release of IL‐1β and exacerbating lung tissue damage [30, 31]. IL‐1β could directly damage the alveolar epithelial barrier and induce the formation of pulmonary edema [32]. And serum IL‐6 levels were positively correlated with lung injury scores and induced inflammatory cell infiltration, forming an “inflammatory cascade effect” [33]. SOD, CAT, and GSH jointly resist oxidative stress by eliminating free radicals and decomposing peroxides; MDA reflects cell membrane oxidative damage; and MPO is a reliable indicator of ALI inflammation severity by damaging lung tissue and amplifying oxidative stress [34]. In summary, TNF‐α, IL‐1β, and IL‐6 form the “core triangle” of the ALI inflammatory cascade, driving neutrophil infiltration and oxidative stress storm. Targeted intervention strategies for ALI need to balance the early blocking of proinflammatory signals and the reconstruction of the redox balance. Fortunately, CY significantly regulated the abnormal levels of these factors, indicating that CY regulated the inflammatory response and oxidative stress in ALI mice.

A number of studies had shown that there was a bidirectional interaction between intestinal barrier function and ALI [35–37]. Goblet cells could secrete mucin and form a physical‐chemical barrier to prevent pathogen invasion. A study has found that pathogens preferentially attack goblet cells, resulting in decreased mucin secretion and barrier disintegration [38]. ZO‐1 and Occludin were central to the maintenance of epithelial permeability [39, 40]. LPS was transposed into the blood after the intestinal barrier and then activated the TLR4 pathway and induced a systemic inflammatory response. Serum DAO and D‐LA levels were increased in patients with ALI, which were positively correlated with the lung injury score [41]. The basic studies had shown that during intestinal infection, LPS entered the blood circulation through the damaged barrier; activated the TLR4 pathway of alveolar macrophages; released TNF‐α, IL‐1β, IL‐6, and other proinflammatory factors; and recruited neutrophils to infiltrate the lungs. In addition, LPS‐induced ALI was accompanied by a burst of reactive oxygen species (ROS), depletion of antioxidant substances (GSH and SOD), and elevation of lipid peroxides (MDA), leading to alveolar epithelial ferroptosis. SIgA is the core component of airway mucosal immunity, which plays a dual role of “immune barrier guardian” and “inflammatory regulatory hub” in ALI. A study has shown that intestinal LPS could destroy alveolar epithelial tight junction and reduce local SIgA secretion after entering the blood, forming a vicious cycle of “gut–lung axis” [42]. A study has confirmed that APS could enhance the synthesis of SIgA and inhibit the release of TNF‐α through the TLR4/NF‐κB pathway, thus achieving the dual regulation of anti‐inflammatory and immunity [43]. Therefore, dynamic detection of the level of SIgA in vivo combined with inflammatory factors could more accurately evaluate the progress and treatment response of ALI. Our results found that LPS could lead to abnormal expression of the above indicators, which were improved by CY intervention, indicating that CY had a good therapeutic effect on intestinal barrier damage and integrity in ALI mice. At the same time, these evidences confirmed the anti‐ALI effect of CY through the gut–lung axis.

The mechanism by which gut microbiota and its metabolites regulate ALI through the “gut–lung” axis has become increasingly clear, and its core lies in the interaction of flora imbalance, metabolite disorder, and immune inflammatory cascade [21]. Therefore, 16S rRNA was applied to deeply explore the mediating role of gut microbiota and their derived metabolites in CY anti‐ALI. CY could balance the abnormal changes in α and β diversity index in ALI mice, which was mainly reflected in the increased abundance of *Ligilactobacillus* and *Parabacteroides* and the decreased abundance of *Lachnospiraceae*. Previous studies had shown that ALI mice were accompanied by a reduction in *unclassified_f_Lachnospiraceae*, *Lachnoclostridium*, and *Facklamia Ileibacterium*. In addition, *Parabacteroides*, *Bacteroides*, and *Escherichia-Shigella* were present in a relatively high abundance in rats with sepsis‐induced ALI. Surprisingly, the levels of these microbiota were effectively altered by CY. These results suggested that CY could improve the symptoms of ALI mice by increasing the diversity of intestinal microecology and correcting the imbalance of the flora structure. As an emerging systems approach, metabolomics adopts a “top‐down” strategy to reflect the function of an organism from the terminal phenotypes of the metabolic network and to analyze the system‐wide metabolic network changes induced by interventions or stimuli in the global context [44]. The focus is on the qualitative and quantitative analysis of metabolites in the whole living system as well as the dynamic response to the changes of endogenous and exogenous factors. At the same time, the derived endogenous metabolites could further amplify and reflect the subtle changes of genes and proteins. Metabolomics, as an emerging discipline in the postgenomics era, is most consistent with the holistic concept of TCM theory, enabling us to better understand the interaction between diseases and metabolites. Both groups of metabolomics results found significant enrichment of unsaturated fatty acid synthesis, proving that it plays a central role in the pathogenesis of ALI and the treatment of CY. For example, intratracheal instillation of γ‐linoleic acid partially inhibited ozone‐induced pulmonary inflammation [45]. Furthermore, nanoparticles constructed from stearic acid could treat ALI [46]. Another study found that arachidonic acid induced lung epithelial cell damage [47]. Further correlation analysis showed that there were different degrees of correlation between γ‐linolenic acid, palmitic acid, linoleic acid, stearic acid, arachidonic acid, 8Z, 11Z, 14z‐eicosatrienoic acid, and *Ligilactobacillus*. In short, the intervention of CY on ALI was likely to be accomplished through the gut microbiota–derived metabolites, and the gut–lung communication was realized.

The Wnt signaling pathway is involved in the adhesion between inflammatory cells and endothelial cells, and it can transform cell phenotype by regulating epithelial interstitial cells and bone marrow mesenchymal cells. Under normal circumstances, the Wnt/β‐catenin pathway is in a static state in adult organs and is activated when stimulated by external stimuli, and the ligand expression of Wnt increases. Further activation of intracellular disheveled protein leads to the inactivation of glycogen synthase kinase‐3β (GSK3β) protein, while β‐catenin protein enters the nucleus and promotes the transcription of the inflammatory factors, which in turn leads to ALI [48, 49]. A study confirmed that bone marrow mesenchymal stem cells that overexpress HOXB4 can improve LPS‐induced ALI by regulating the Wnt/β‐catenin signaling pathway [50]. Shensiqigui tablets also alleviated pulmonary fibrosis though acting Hedgehog/Wnt/β‐catenin [51]. Besides, knocking out the RAB6 gene can activate Wnt/β‐catenin, which plays an indispensable role in alleviating lung injury induced by particulate matter (PM2.5) and delaying the progression of fibrosis [52]. A study confirmed that miR‐600 targets stearoyl desaturase1 (SCD1) can regulate the Wnt signaling pathway by synthesizing unsaturated fatty acids [53]; Jianpi Yangzheng Xiaozheng granule may inhibit the synthesis of unsaturated fatty acids by regulating the SCD1/Wnt/β‐catenin axis, therefore inhibiting the occurrence of poorly cohesive gastric cancer [54]. Our investigation confirmed that CY could significantly inhibit the Wnt/β‐catenin pathway, which is specifically manifested as downregulating Wnt and β‐catenin proteins, activating the GSK3β protein, and promoting its phosphorylation.

**Figure 11:**
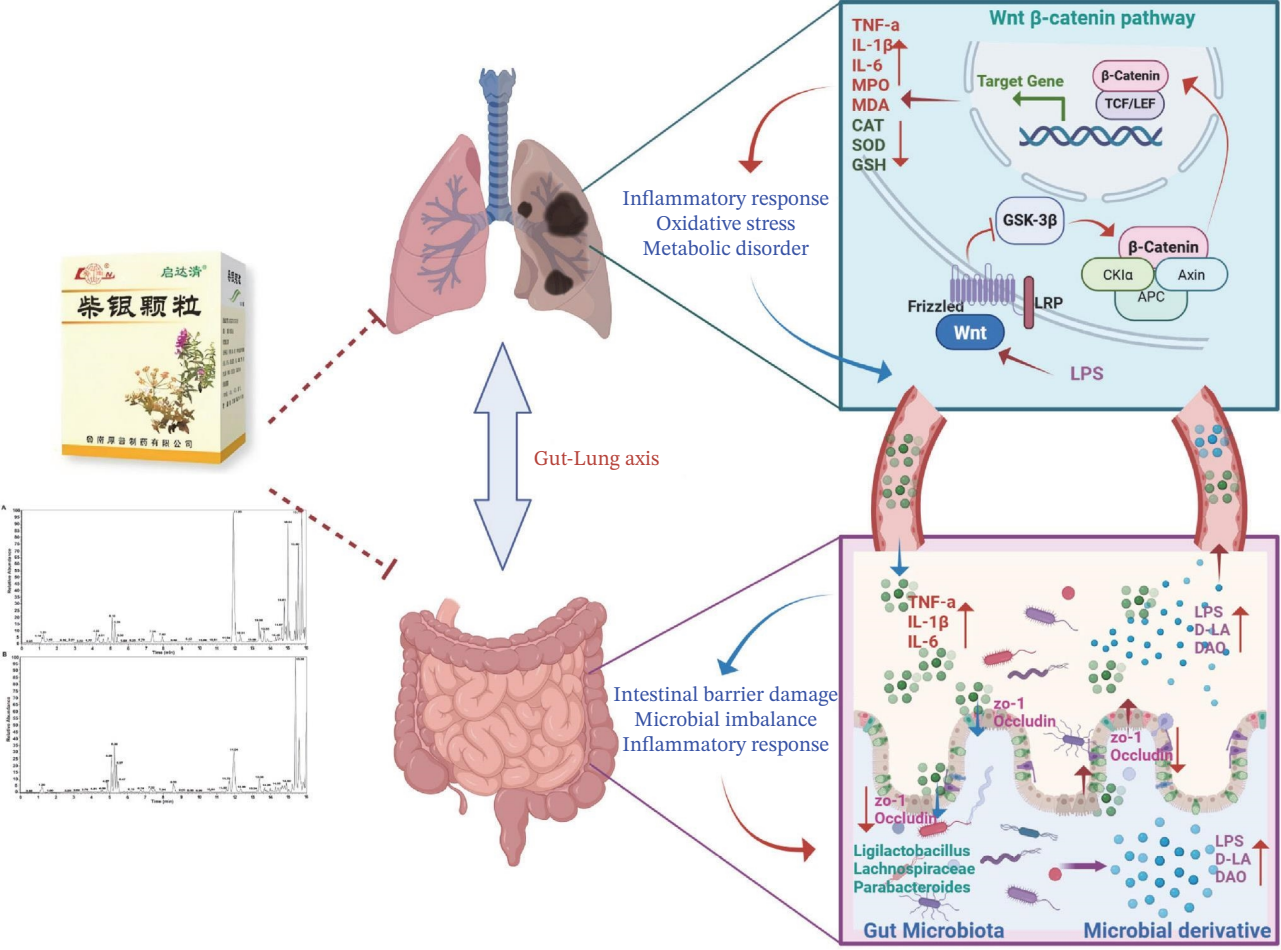
The protective mechanism network of CY against LPS‐induced ALI in mice.

In summary, the multiomics data results revealed that CY achieved multilevel synergistic anti‐ALI effects through the gut–lung axis (Figure 11). A potential regulatory mechanism can be summarized as follows: CY reversed the intestinal flora disorder induced by ALI, restored the abundance of key genera, and reduced the entry of intestinal toxins (LPS, D‐LA, and DAO) into the blood. At the metabolic level, CY regulated the dysregulated metabolite profiles in serum and lung tissues, especially enriching pathways such as unsaturated fatty acid biosynthesis, and the key metabolites were significantly correlated with the above core flora, suggesting that the gut microbiota regulated by CY may mediate gut–lung axis communication through regulating metabolites. Further, at the protein level, CY inhibited the abnormal activation of the Wnt signaling pathway (Wnt3a/β‐catenin) in lung tissue induced by LPS, ultimately exerting a protective effect on lung tissue cells. In short, the pharmacological effect is the final phenotype of multiomics regulation. The above changes jointly mediate CY’s improvement of lung injury, alleviation of inflammation and oxidative stress, and protection of the intestinal barrier, forming a complete regulatory chain of “microbiota structure remodeling → metabolite‐mediated gut‐lung axis communication → protein pathway (Wnt) regulation → multi‐dimensional pharmacological output,” thereby revealing the mechanism of CY’s synergistic anti‐ALI effect through the gut–lung axis.

This study has provided a new target and research strategy for the treatment of ALI with CY. However, there are still some deficiencies in this study. The verification of the causal relationship of intestinal flora, the intervention of metabolites, and the specific molecular mechanism still needs to be further explored. Besides, our study does not consider the individual differences of other species or clinical patients. Therefore, fecal microbiota transplantation, gene knockout, and other means can be used in the future, as well as large‐sample studies in the population, to jointly and thoroughly clarify the specific effects and actual efficacy of CY.

## 5. Conclusion

Based on the multiomics integration study, combined with the theory of gut–lung axis, the present study elucidated that CY could effectively improve LPS‐induced inflammation and oxidative stress in ALI mice by inhibiting the activation of the Wnt/β‐catenin signaling pathway to regulate gut microbiota and metabolism disorders. This systematic pharmacology strategy not only reveals a new mechanism of CY in the treatment of ALI but also provides a new research direction for the development of innovative therapeutic drugs for ALI.

## Author Contributions

**Wenting Ni**: writing – original draft, visualization, methodology, validation. **He Xiao and Qun Feng**: data curation, supervision, resources, methodology. **Shirong Li, Jingchun Yao, and Guimin Zhang**: writing – review and editing, funding acquisition, project administration.

## Funding

This work was granted by the Natural Science Foundation of Shandong Province (Joint Foundation for Innovation and Development) (Grant ZR2022LZY021).

## Conflicts of Interest

The authors declare no conflicts of interest.

## Supporting Information

Additional supporting information can be found online in the Supporting Information section.

## Supporting information


**Supporting Information** Supporting Information 1 Table S1: Key differentially expressed serum metabolites. Supporting Information 2 Table S2: Key differentially expressed lung metabolites.

## Data Availability

Data will be made available upon request.
