# Supplementary material for: Chaiyin Granules Improve Acute Lung Injury by Regulating Gut Microbiota and Unsaturated Fatty Acid Mediated Wnt/β‐Catenin Signaling Pathway
Source: J Immunol Res. 2026 Jun 3;2026:7225946. doi: 10.1155/jimr/7225946 (PMC13581059; doi:10.1155/jimr/7225946)
Supplement: Supplementary file 1 [file JIMR-2026-7225946-s001.docx]

**Supplementary material 1**

Table 1 Key differentially expressed serum metabolites

|  | Metabolite | Formula | m/z | RT/min | VIP值 | ESI Mode | Variation trend | |  |  |
| --- | --- | --- | --- | --- | --- | --- | --- | --- | --- | --- |
| No. |  |  |  |  |  |  | Model vs Control | CYH vs Model | p value(Control vs Model) | p value(Model vs CYH) |
| 1 | Caffeic acid 3-sulfate | C9H8O7S | 258.99168 | 3.552 | 1.05676 | - | ↑** | ↓** | 0.0007295 | 0.0004441 |
| 2 | N-[(15Z)-tetracosenoyl]sphing-4-enine-1-phosphocholine | C47H93N2O6P | 813.68092 | 10.151 | 1.21973 | + | ↑** | ↓** | 9.832E-08 | 0.0007217 |
| 3 | 1_6_6-Trimethyl-2_7-dioxabicyclo[3.2.2]nonan-3-one | C10H16O3 | 183.10188 | 5.534 | 1.43044 | - | ↓** | ↑** | 5.671E-06 | 0.002226 |
| 4 | Linoleyl carnitine | C25H45NO4 | 424.34132 | 8.584 | 3.10231 | + | ↑** | ↓** | 9.374E-08 | 0.0029915 |
| 5 | L-Tyrosine | C9H11NO3 | 182.08119 | 1.476 | 2.48855 | + | ↑** | ↓** | 6.828E-05 | 0.0034553 |
| 6 | Hexamethylenetetramine | C6H12N4 | 141.11357 | 0.398 | 2.68159 | + | ↓** | ↑** | 2.344E-06 | 0.0039725 |
| 7 | (2E)-hexadecenoylcarnitine | C23H43NO4 | 398.32586 | 8.242 | 1.91717 | + | ↑** | ↓** | 2.872E-05 | 0.004601 |
| 8 | C14-Carnitine | C21H41NO4 | 372.31062 | 7.889 | 1.9565 | + | ↑** | ↓** | 5.757E-06 | 0.0060522 |
| 9 | 11,12-Epoxy-(5Z,8Z,11Z)-icosatrienoic acid | C20H32O3 | 319.22769 | 8.83 | 3.842 | - | ↑** | ↓** | 0.0004503 | 0.0085542 |
| 10 | DL-Arginine | C6H14N4O2 | 175.1191 | 13.442 | 1.72566 | + | ↓** | ↑** | 1.042E-06 | 0.0089338 |
| 11 | 14(S)-HDHA | C22H32O3 | 343.22769 | 8.709 | 2.33175 | - | ↑** | ↓** | 0.0005365 | 0.0092139 |
| 12 | Docosapentaenoic acid | C22H34O2 | 329.24847 | 10.573 | 1.54653 | - | ↑** | ↓** | 0.0014068 | 0.0092491 |
| 13 | (+/-)12-HpETE | C20H32O4 | 317.21199 | 8.383 | 1.98709 | - | ↑** | ↓** | 0.0013111 | 0.0094366 |
| 14 | 3-tert-Butyladipic acid | C10H18O4 | 225.1098 | 4.04 | 2.08487 | + | ↑** | ↓* | 0.0003975 | 0.0104079 |
| 15 | 3,4-Dihydroxyphenylpropionic acid | C9H10O4 | 165.05464 | 1.478 | 1.117 | + | ↑* | ↓* | 0.0116031 | 0.0115437 |
| 16 | Aliskiren | C30H53N3O6 | 552.40159 | 8.007 | 1.71894 | + | ↓* | ↑* | 0.0115993 | 0.012107 |
| 17 | D-γ-Glutamyl-S-[(5Z,8β,12E,15S)-1,15-dihydroxy-1,11-dioxoprosta-5,12-dien-9-yl]-L-cysteinylglycine | C30H47N3O10S | 640.29219 | 9.635 | 1.62527 | - | ↓* | ↑* | 0.0325134 | 0.012862 |
| 18 | (2R)-1-[(Hydroxy{[(1s,3R)-2,3,4,5,6-pentahydroxycyclohexyl]oxy}phosphoryl)oxy]-3-(stearoyloxy)-2-propanyl (5Z,8Z,11Z,14Z)-5,8,11,14-icosatetraenoate | C47H83O13P | 885.55041 | 10.638 | 4.29724 | - | ↓** | ↑* | 0.0005339 | 0.0146108 |
| 19 | γ-Linolenic acid | C18H30O2 | 277.21707 | 10.07 | 5.11429 | - | ↑** | ↓* | 0.0090522 | 0.014953 |
| 20 | N-Benzylformamide | C8H9NO | 136.07568 | 1.48 | 1.5479 | + | ↑** | ↓* | 5.379E-05 | 0.015054 |
| 21 | 9,12-Hexadecadienoylcarnitine | C23H41NO4 | 396.31062 | 7.674 | 1.42781 | + | ↑** | ↓* | 7.395E-06 | 0.0163235 |
| 22 | [FA(18:4)]6Z_9Z_12Z_15Z-octadecatetraenoicacid | C18H28O2 | 275.20142 | 9.6 | 1.57744 | - | ↑* | ↓* | 0.0100907 | 0.0180129 |
| 23 | 7Z,10Z-Hexadecadienoic acid | C16H28O2 | 251.20135 | 9.801 | 2.71376 | - | ↑** | ↓* | 0.0003221 | 0.0195075 |
| 24 | Palmitoleic Acid | C16H30O2 | 253.21703 | 10.398 | 6.93539 | - | ↑* | ↓* | 0.0209981 | 0.0195628 |
| 25 | Taurocholate | C26H45NO7S | 514.28419 | 5.478 | 1.12507 | - | ↑** | ↓* | 0.0038788 | 0.0215055 |
| 26 | 1-(Dodecanoyloxy)-3-(octanoyloxy)-2-propanyl myristate | C37H70O6 | 609.51 | 10.732 | 1.82657 | - | ↑** | ↓* | 0.0047764 | 0.0247522 |
| 27 | NP-008915 | C15H24O3 | 233.15427 | 8.942 | 1.10541 | - | ↑** | ↓* | 0.0008906 | 0.026107 |
| 28 | (9Z)-Tetradecenoicacid | C14H26O2 | 225.18549 | 9.574 | 2.60403 | - | ↑** | ↓* | 0.0024993 | 0.0262837 |
| 29 | 2,6-di-tert-Butylphenol | C14H22O | 205.1591 | 9.4 | 2.43863 | - | ↑* | ↓* | 0.0196965 | 0.0283359 |
| 30 | Oleoylcarnitine | C25H47NO4 | 426.35697 | 9.349 | 2.80729 | + | ↑** | ↓* | 2.433E-05 | 0.028356 |
| 31 | D-(+)-Mannose | C6H12O6 | 179.05526 | 0.751 | 1.82595 | - | ↓** | ↑* | 3.522E-05 | 0.0297345 |
| 32 | Methylfarnesoate | C16H26O2 | 249.18568 | 9.38 | 1.4932 | - | ↑** | ↓* | 0.0013392 | 0.0297731 |
| 33 | 2-Amino-1,3,4-octadecanetriol | C18H39NO3 | 318.30018 | 7.459 | 1.69422 | + | ↑* | ↓* | 0.0387075 | 0.0297897 |
| 34 | Lauric acid | C12H24O2 | 199.16962 | 9.204 | 1.7157 | - | ↑** | ↓* | 0.0034989 | 0.0306569 |
| 35 | Palmitic Acid | C16H32O2 | 255.23274 | 11.188 | 4.12333 | - | ↑** | ↓* | 0.0097887 | 0.0350466 |
| 36 | Pantothenate | C9H17NO5 | 220.118 | 2.865 | 2.49497 | + | ↑** | ↓* | 1.02E-07 | 0.0352603 |
| 37 | trans-10-Heptadecenoic acid | C17H32O2 | 267.23283 | 10.866 | 1.01769 | - | ↑* | ↓* | 0.0415136 | 0.0394839 |
| 38 | cis-5-Tetradecenoylcarnitine | C21H39NO4 | 370.29525 | 7.385 | 1.46648 | + | ↑** | ↓* | 0.0006706 | 0.0406028 |
| 39 | 4-Hydroxybenzaldehyde | C7H6O2 | 123.04419 | 1.478 | 1.07363 | + | ↑** | ↓* | 7.848E-05 | 0.0407885 |
| 40 | 2-C-Methyl-D-erythritol4-phosphate | C5H13O7P | 215.03224 | 0.751 | 2.20095 | - | ↓** | ↑* | 0.0001727 | 0.0419622 |
| 41 | Linoleic acid | C18H32O2 | 279.23275 | 10.611 | 8.60769 | - | ↑** | ↓* | 0.0012711 | 0.0440449 |
| 42 | Methionine | C5H11NO2S | 150.05833 | 1.132 | 1.91333 | + | ↑** | ↓* | 0.0001239 | 0.0458061 |

**p*＜0.05，***p*＜0.1.

**Supplementary material 2**

Table 2 Key differentially expressed lung metabolites

|  | Metabolite | Formula | m/z | RT/min | VIP值 | ESI Mode | Variation trend | |  |  |
| --- | --- | --- | --- | --- | --- | --- | --- | --- | --- | --- |
| No. |  |  |  |  |  |  | Model vs Control | CYH vs Model | p value (Model vs Control) | p value (CYH VS Model ) |
| 1 | 1-Palmitoyl-2-(5-keto-8-oxo-6-octenoyl)-sn-glycero-3-phosphatidylcholine | C32 H58 N O10 P | 648.386 | 9.373 | 1.76468 | + | ↑** | ↓** | 9.17E-05 | 0.000137 |
| 2 | DL-Arginine | C6 H14 N4 O2 | 175.119 | 13.523 | 1.49484 | + | ↓** | ↑** | 2.5E-07 | 0.000205 |
| 3 | 1-Palmitoyl-2-(5-hydroxy-8-oxo-6-octenoyl)-sn-glycero-3-phosphatidylcholine | C32 H60 N O10 P | 650.4016 | 9 | 2.40121 | + | ↑** | ↓** | 0.000143 | 0.000237 |
| 4 | N-(5-acetamidopentyl)acetamide | C9 H18 N2 O2 | 187.144 | 4.104 | 1.48676 | + | ↑** | ↓** | 0.000207 | 0.000273 |
| 5 | Isocitric acid | C6 H8 O7 | 191.0191 | 13.386 | 2.90565 | - | ↓** | ↑** | 1.77E-06 | 0.000305 |
| 6 | O-[{(2R)-2,3-Bis[(9Z)-9-tetradecenoyloxy]propoxy}(hydroxy)phosphoryl]-L-serine | C34 H62 N O10 P | 676.4172 | 9.263 | 2.21447 | + | ↑** | ↓** | 0.000197 | 0.000329 |
| 7 | 8Z,11Z,14Z-Eicosatrienoic acid | C20 H34 O2 | 305.2487 | 11.111 | 4.53582 | - | ↓** | ↑** | 0.000121 | 0.000376 |
| 8 | O-{Hydroxy[(2R)-2-[(9Z,12Z,15Z)-9,12,15-octadecatrienoyloxy]-3-(tetradecanoyloxy)propoxy]phosphoryl}-L-serine | C38 H68 N O10 P | 730.4659 | 10.198 | 1.10083 | + | ↑** | ↓** | 0.000382 | 0.00048 |
| 9 | 1,2-Dilauroyl-sn-glycero-3-PE | C29 H58 N O8 P | 580.3963 | 9.649 | 1.12437 | + | ↑** | ↓** | 0.000617 | 0.000819 |
| 10 | Adrenic acid | C22 H36 O2 | 331.2644 | 11.358 | 3.58153 | - | ↓** | ↑** | 1.25E-05 | 0.000886 |
| 11 | 1-Palmitoyl-2-(5-hydroxy-8-oxo-6-octenedioyl)-sn-glycero-3-phosphatidylcholine | C32 H60 N O11 P | 666.3965 | 9.14 | 1.01026 | + | ↑** | ↓** | 0.000821 | 0.000907 |
| 12 | 4-Dodecylbenzenesulfonic acid | C18 H30 O3 S | 325.1843 | 10.744 | 1.74048 | - | ↑** | ↓** | 2.26E-06 | 0.000991 |
| 13 | PAF C-18:1 | C28 H56 N O7 P | 550.3858 | 9.972 | 1.45634 | + | ↓** | ↑** | 6.33E-07 | 0.00105 |
| 14 | Uric acid | C5 H4 N4 O3 | 167.0202 | 1.209 | 2.71294 | - | ↓* | ↑** | 0.043819 | 0.001056 |
| 15 | O-{Hydroxy[(2R)-2-[(5Z,8Z,11Z,14Z)-5,8,11,14-icosatetraenoyloxy]-3-(tetradecanoyloxy)propoxy]phosphoryl}-L-serine | C40 H70 N O10 P | 756.4811 | 10.478 | 1.20789 | + | ↑** | ↓** | 0.000787 | 0.001106 |
| 16 | Cytidine 5'-monophosphate (hydrate) | C9 H14 N3 O8 P | 324.0589 | 1.213 | 1.36274 | + | ↑* | ↓** | 0.010746 | 0.001484 |
| 17 | 3'-CMP | C9 H14 N3 O8 P | 324.0584 | 0.927 | 1.48817 | + | ↑* | ↓** | 0.014965 | 0.001683 |
| 18 | Octodrine | C8 H19 N | 130.159 | 4.155 | 3.6502 | + | ↓* | ↑** | 0.014911 | 0.001724 |
| 19 | Thiamine | C12 H16 N4 O S | 265.1116 | 2.811 | 1.42443 | + | ↓** | ↑** | 0.003216 | 0.00179 |
| 20 | Xanthine | C5 H4 N4 O2 | 151.0252 | 2.006 | 2.57694 | - | ↓** | ↑** | 0.000228 | 0.002204 |
| 21 | 11(Z),14(Z)-Eicosadienoic Acid | C20 H36 O2 | 307.2643 | 11.824 | 3.12047 | - | ↓** | ↑** | 0.000926 | 0.002531 |
| 22 | Prostaglandin I2 | C20 H32 O5 | 353.2319 | 5.38 | 1.61829 | + | ↓** | ↑** | 3.04E-05 | 0.003604 |
| 23 | N-Nitrosoproline | C5 H8 N2 O3 | 145.0607 | 1.512 | 1.97468 | + | ↓* | ↑** | 0.009701 | 0.003712 |
| 24 | N-Stearoyltyrosine | C27 H45 N O4 | 448.3414 | 8.722 | 2.0148 | + | ↓** | ↑** | 0.000223 | 0.004477 |
| 25 | all-cis-4,7,10,13,16-Docosapentaenoic acid | C22 H34 O2 | 329.2486 | 10.96 | 2.95357 | - | ↓* | ↑** | 0.012185 | 0.005699 |
| 26 | O-(Hydroxy{(2R)-3-(tetradecanoyloxy)-2-[(9Z)-9-tetradecenoyloxy]propoxy}phosphoryl)-L-serine | C34 H64 N O10 P | 678.433 | 10.084 | 1.57195 | + | ↑** | ↓** | 0.004548 | 0.006556 |
| 27 | D-Carnitine | C7 H15 N O3 | 162.1123 | 1.202 | 1.57597 | + | ↓** | ↑** | 6.96E-06 | 0.007651 |
| 28 | 5-[(1S,2R,4aR)-5-(Hydroxymethyl)-1,2,4a-trimethyl-1,2,3,4,4a,7,8,8a-octahydro-1-naphthalenyl]-3-methylpentanoic acid | C20 H34 O3 | 321.2436 | 9.237 | 1.37065 | - | ↓** | ↑** | 6.84E-05 | 0.008734 |
| 29 | 12(S)-HETE | C20 H32 O3 | 319.2279 | 8.992 | 4.28803 | - | ↓** | ↑** | 7.93E-07 | 0.009109 |
| 30 | Hypoxanthine | C5 H4 N4 O | 137.0457 | 2.905 | 4.33566 | + | ↓** | ↑* | 0.00154 | 0.009962 |
| 31 | 14(S)-HDHA | C22 H32 O3 | 343.2279 | 8.871 | 1.63031 | - | ↓** | ↑* | 0.00121 | 0.01313 |
| 32 | 1-[(11Z,14Z)]-icosadienoyl-sn-glycero-3-phosphocholine | C28 H54 N O7 P | 548.3701 | 9.187 | 1.28468 | + | ↓** | ↑* | 7.14E-07 | 0.013299 |
| 33 | Dioleoylphosphatidylserine | C42 H78 N O10 P | 788.5418 | 11.434 | 2.11535 | + | ↑* | ↓* | 0.017102 | 0.013525 |
| 34 | Arachidonic acid | C20 H32 O2 | 303.2329 | 10.646 | 6.97585 | - | ↓** | ↑* | 0.005355 | 0.013629 |
| 35 | Stearic acid | C18 H36 O2 | 283.2643 | 12.822 | 2.88675 | - | ↓** | ↑* | 1.41E-05 | 0.014418 |
| 36 | Ethyl docosahexaenoate | C24 H36 O2 | 355.2644 | 11.06 | 1.08132 | - | ↓** | ↑* | 2.23E-05 | 0.015385 |
| 37 | [(4Z)-5-(Methylsulfanyl)-4-penten-2-yn-1-yl]benzene | C12 H12 S | 189.0735 | 13.403 | 1.65256 | + | ↓** | ↑* | 0.006776 | 0.015421 |
| 38 | Caprolactam | C6 H11 N O | 114.0915 | 0.327 | 1.62025 | + | ↓** | ↑* | 9.17E-05 | 0.017 |
| 39 | Taurine | C2 H7 N O3 S | 124.0063 | 0.784 | 2.56266 | - | ↓* | ↑* | 0.039995 | 0.017227 |
| 40 | O-[{(2R)-2-[(9Z)-9-Hexadecenoyloxy]-3-[(9Z)-9-tetradecenoyloxy]propoxy}(hydroxy)phosphoryl]-L-serine | C36 H66 N O10 P | 704.4487 | 10.155 | 1.46389 | + | ↑* | ↓* | 0.016767 | 0.019173 |
| 41 | 2-Formylglutarate | C6 H8 O5 | 199.001 | 13.737 | 3.13962 | + | ↑** | ↓* | 0.000265 | 0.019193 |
| 42 | 2-Oxo-3-(phosphonooxy)propyl formate | C4 H7 O7 P | 199.001 | 13.737 | 3.13962 | + | ↑** | ↓* | 0.000265 | 0.019193 |
| 43 | 4-Oxoproline | C5 H7 N O3 | 128.0342 | 0.893 | 1.83112 | - | ↓* | ↑* | 0.040107 | 0.022174 |
| 44 | PC(P-16:0/16:0) | C40 H80 N O7 P | 718.5726 | 10.595 | 3.31323 | + | ↑** | ↓* | 0.003996 | 0.02432 |
| 45 | Glycerophospho-N-palmitoyl ethanolamine | C21 H44 N O7 P | 452.2784 | 8.608 | 1.19149 | - | ↓** | ↑* | 0.000961 | 0.025235 |
| 46 | 6-Ketoprostaglandin F1α | C20 H34 O6 | 369.2284 | 5.382 | 1.0588 | - | ↓** | ↑* | 2.15E-05 | 0.026418 |
| 47 | Choline | C5 H13 N O | 104.1072 | 0.857 | 7.78542 | + | ↓** | ↑* | 5.73E-05 | 0.026565 |
| 48 | Linoleamide | C18 H33 N O | 280.2629 | 9.438 | 1.21861 | + | ↓** | ↑* | 1.1E-06 | 0.030711 |
| 49 | asulam | C8 H10 N2 O4 S | 231.0434 | 3.518 | 1.59269 | + | ↓** | ↑* | 7.08E-05 | 0.030921 |
| 50 | 1-(1Z-hexadecenyl)-sn-glycero-3-phosphocholine | C24 H50 N O6 P | 480.3441 | 8.965 | 1.66299 | + | ↓** | ↑* | 1.36E-05 | 0.032397 |
| 51 | 1-Oleoylglycerophosphocholine | C26 H52 N O7 P | 522.3551 | 8.934 | 6.08374 | + | ↓** | ↑* | 6.33E-05 | 0.034398 |
| 52 | LysoPC(22:4(7Z,10Z,13Z,16Z)) | C30 H54 N O7 P | 572.3703 | 9.031 | 1.31489 | + | ↓** | ↑* | 2.82E-05 | 0.037081 |
| 53 | PG(16:1(9Z)/20:4(5Z,8Z,11Z,14Z)) | C42 H73 O10 P | 767.4872 | 10.689 | 1.72882 | - | ↓* | ↑* | 0.021162 | 0.040679 |
| 54 | Xaliproden | C24 H22 F3 N | 382.1791 | 3.558 | 1.34447 | + | ↑** | ↓* | 0.005747 | 0.041334 |
| 55 | Adenosine 5'-monophosphate | C10 H14 N5 O7 P | 348.0697 | 1.707 | 1.35654 | + | ↑* | ↓* | 0.033641 | 0.043852 |
| 56 | Uridine monophosphate (UMP) | C9 H13 N2 O9 P | 323.0287 | 0.927 | 1.70148 | - | ↑* | ↓* | 0.018868 | 0.046115 |
| 57 | PG(16:0/22:5(7Z,10Z,13Z,16Z,19Z)) | C44 H77 O10 P | 795.5184 | 10.909 | 3.2912 | - | ↓** | ↑* | 0.003191 | 0.046711 |
| 58 | PC | C52 H80 N O8 P | 878.5684 | 10.733 | 1.15456 | + | ↓* | ↑* | 0.02305 | 0.049457 |

**p*＜0.05，***p*＜0.1.
